# Environmentally friendly chitosan/PEI-grafted magnetic gelatin for the highly effective removal of heavy metals from drinking water

**DOI:** 10.1038/srep43082

**Published:** 2017-02-22

**Authors:** Bingbing Li, Feng Zhou, Kai Huang, Yipei Wang, Surong Mei, Yikai Zhou, Tao Jing

**Affiliations:** 1 State Key Laboratory of Environment Health (Incubation), Key Laboratory of Environment and Health, Ministry of Education, Key Laboratory of Environment and Health (Wuhan), Ministry of Environmental Protection, School of Public Health, Tongji Medical College, Huazhong University of Science and Technology, #13 Hangkong Road, Wuhan, Hubei, 430030, China; 2 Institute of Environmental Pollution and Health, School of Environmental and Chemical Engineering, Shanghai University, Shanghai 200444, China

## Abstract

The development of environmentally friendly sorbents with a high adsorption capacity is an essential problem in the removal of heavy metals from drinking water. In this study, magnetic gelatin was prepared using transglutaminase as a cross-linker, which could only catalyze an acyl-transfer reaction between lysine and glutamine residues of the gelatin and not affect other amino groups. Therefore, it was beneficial for the further modification based on the amino groups, and did not affect the spatial structure of gelatin, which can effectively prevent the embedding of active sites in the polymer matrix. After modification with the chitosan/polyethylenimine copolymers, the numbers of amino groups was greatly increased, and the magnetic composites exhibited a high adsorption capacity, excellent water compatibility and simple magnetic separation. The adsorption capacities of lead and cadmium were 341 mg g^−1^ and 321 mg g^−1^, respectively, which could be used for the removal of metal ions in drinking water.

Numerous health-related pollution incidents, especially for the heavy metal pollution, have raised public concern in the world due to their toxicity, persistence and bio-accumulative nature. Long-term consumption of drinking water containing heavy metals can result in serious health threat, such as neurological, cardiovascular, renal, gastrointestinal, haematological and reproductive effects^1^. The current regulatory limits of the U.S. environmental protection agency (EPA) for lead (Pb) and cadmium (Cd) are 15 ng mL^−1^ and 5 ng mL^−1^ in drinking water, respectively; whereas the WHO sets a lower limit of 10 ng mL^−1^ for Pb(II) ions. The U.S. EPA classified Cd as a human carcinogen at low levels of exposure. Levallois reported that the pipe materials and fittings in contact with drinking water are important sources of lead exposure^2^. To date, there is no risk-free level of exposure to lead that has been found. Although children with blood lead level <10 μg dL^−1^ are not be identified as “lead poisoning”, low-level environmental exposure (<7.5 μg dL^−1^) is still linked to substantial neuro-behavioral problems in children^3^,^4^. Therefore, an efficient, cost-effective, and practical technology is urgent for the decontamination of water without endangering human health.

Great efforts have been made to explore various water treatment techniques to remove heavy metals, such as adsorption, chemical precipitation, membrane separation and electrochemical technologies. In these strategies, adsorption offers flexibility in its operation and a low operating cost. However, the removal efficiencies of heavy metals were in the range of 6–35% by using most commercial water purifiers. Thus, various sorbents, such as carbon-based materials (activated carbon^5^,^6^, carbon nanotubes^7^,^8^ and graphene^9^), nanosized metal oxides (manganese oxides^10^,^11^) and chitosan composites, are all considered in the literatures to be promising choices for the effective adsorption of heavy metals. Although the large surface areas of these nanomaterials lead to high adsorption capacities, the difficulty of recovering the materials and the high pressure of the packed column are inevitable. Furthermore, it is important to consider that trace residual carbon-based materials in drinking water are toxic and may become a new class of hazardous pollutants to threat public health^12^,^13^. Khan suggested that residual manganese in drinking water is a potential threat to children’s health^14^. Thus, chitosan or gelatin materials are considered to be promising choices for effective removing heavy metals from drinking water^15^. Unfortunately, the application of chitosan materials was unsatisfactory due to poor solubility and pKa value (about 6.3–6.4).

Gelatin is a traditional water-soluble biopolymer with the distinctive advantages of nontoxicity, biodegradability, and low cost, and it can act as a sorbent to form complexes with metal ions^16^,^17^. However, the amino groups of gelatin (or chitosan) can not only be used as the crosslinking groups to maintain the mechanical stability but also as the main recognition sites to guarantee the removal efficiency of the sorbents. The results of Gyananath and Balhal also verify the effect of the cross-linker on the removal efficiency of Pb(II) using chitosan materials. The adsorption capacity was 39.42 mg g^−1^ on the chitosan beads cross-linked, which was much lower than that without cross-linking (72.89 mg g^−1^)^18^. It is shown that the adsorption capacity of sorbents prepared by gelatin or chitosan as a monomer is low, which restricts its practical application, especially for the removal of heavy metals with low concentrations in drinking water. Furthermore, conventionally used cross-linkers were carbodiimides, polyepoxy compounds and aldehydes. The main shortcoming was the toxic effects caused by residual cross-linker^19^. Transglutaminase (TGases) can be used to catalyze an acyl-transfer reaction between the ε-amino groups (lysine residues) and γ-carboxamide groups (glutamine residues), resulting in the formation of ε-(γ-glutamyl) lysine isopeptide bonds^20^. The TGase is environmental friendly and the cross linking procedure is only performed between Lys and Gln without involving other amino acid. Therefore, while maintaining the mechanical stability, lots of amino groups remained for the highly efficient adsorption of heavy metals.

In this study, magnetic gelatin was prepared in an easy and effective way using TGases as a cross linker, and it exhibited a high adsorption capacity, good dispersion and high magnetization. To further improve the adsorption capacity and the mechanical stability, chitosan grafted polyethylenimine (PEI) cationic copolymers are synthesized and then grafted onto the surface of magnetic gelatin. Subsequently, it was used as a sorbent to remove heavy metals from drinking water at a low initial concentration and the removal properties were evaluated through the adsorption isotherm and adsorption kinetics. The results indicated that the magnetic composites have a wide range of applications in water purification treatment.

## Results and Discussion

### Preparation and characterizations of chitosan/PEI-grafted magnetic gelatin

**Figure 1:**
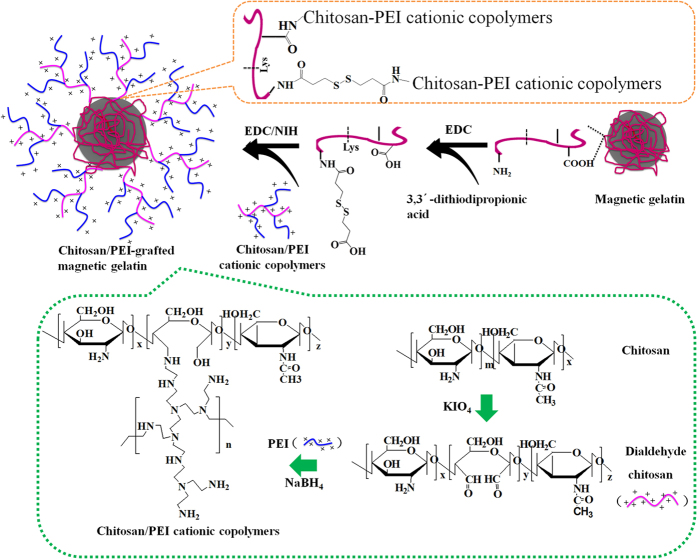
Preparation procedure of the chitosan/PEI-grafted magnetic gelatin.

In this study, preparation of the chitosan/PEI-grafted magnetic gelatin is shown in Fig. 1. Although nanomaterials exhibited excellent adsorption performance, the difficulty of recovering materials and high pressure of packed columns were inevitable, which would affect its practical application in the removal of heavy metals. Therefore, considerable attention has been paid to magnetic nanomaterials for their fast and feasible separation. First, magnetic gelatin was prepared by an easy and effective method. Because gelatin had a net negative surface charge in an ammonia solution, the iron ions could be distributed in the gelatin by electrostatic interactions and then formed the iron oxide nanoparticles in a basic solution at a high temperature. After magnetic separation, the magnetic gelatin was further cross-linked by TGase to maintain its mechanical stability. Compared with other cross-linkers, the most important property of TGase in this study was the substrate specificity (Lys-Gln). Therefore, lots of amino groups could be remained for further modification and removal of heavy metals in drinking water^20^. The cross-linking mechanism is shown in Fig. S1 (Supporting Information).

Subsequently, chitosan/PEI cationic copolymers were synthesized and modified onto the surface of the magnetic composite to further improve the adsorption capacity. Periodate oxidation was beneficial for the flexibility and solubility of chitosan. PEI was grafted onto chitosan to form a cationic copolymer, which could further improve adsorption capacity of the resulting polymer and its stability in strong alkaline or acidic solution. The grafted efficiency of PEI in the cationic copolymers was about 38.7%, based on the evaluated results of 2,4,6-trinitrobenzenesulfonic acid reaction^21^. In Fig. S2, chitosan was oxidatively cleaved and a new peak was appeared at 2.9 ppm, corresponding to the formation of chitosan/PEI copolymer (Supporting Information).

In this study, 3,3′-dithiodipropionic acid with two carboxylic groups (-COOH) was grafted to the magnetic gelatin due to the reaction between the carboxylic groups and the amino groups of gelatin. The degree of amino substitution was determined by the 2,4,6-trinitrobenzenesulfonic acid reaction to study the grafted efficiency and it was about 79.4%. Thus, the surface of magnetic gelatin possessed abundant carboxylic groups. Then, chitosan-PEI cationic copolymers were further modified on the magnetic gelatin using EDC/NHS coupling to obtain the target product chitosan/PEI-grafted magnetic gelatin and the number of amino groups was significantly increased. The amino groups played a primary role to remove the heavy metals in neutral solutions (drinking water) due to the formation of chelation complexes. Thus, transglutaminase as a cross-linker and the further modification of cationic copolymers were used to improve the number of amino groups and enhance the removal efficiency. Furthermore, the gelatin could also form complexes with metal ions through various side groups such as imidazole and thiol.

**Figure 2:**
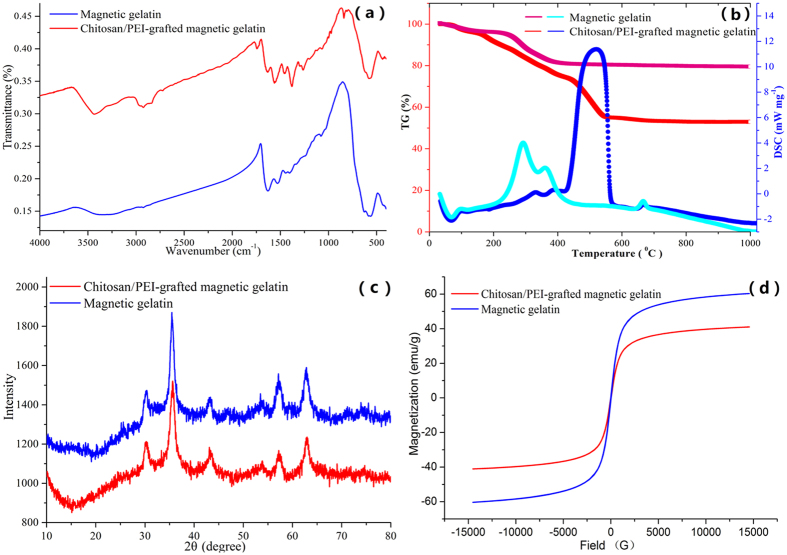
(**a**) FTIR spectra, (**b**) XRD patterns, (**c**) thermal analyses and (**d**) hysteresis loop of magnetic gelatin and chitosan/PEI-grafted magnetic gelatin.

Figure S3 (Supporting Information) shows the sizes and morphologies of the magnetic gelatin and chitosan/PEI-grafted magnetic gelatin. From the TEM images, we can see that the magnetic gelatin was homogeneously dispersed with a mean diameter of 15 nm. After being grafted with chitosan/PEI cationic copolymers, the magnetic gelatin formed a monolayer sheet structure with a mean diameter of 500 nm. The results indicated that there was a cross-linking between one chitosan/PEI copolymer (-NH_3_) and multiple magnetic gelatin particles (-COOH). However this procedure was only performed in a plane due to the repulsion between positively charged amino groups. To verify the combination of magnetic composites, FTIR spectra were collected as shown in Fig. 2a. The characteristic bands of iron oxide nanoparticles are 572 and 447 cm^−1^. The characteristic bands at 1643 and 1542 cm^−1^ belonged to the amide I and amide II of gelatin, respectively, as well as the δ(N-H) of primary amines at 3234 cm^−1^. The broad band due to C-H stretching vibrations occurred in the region from 2700 to 3000 cm^−1^, C=O stretching occurred at 1775 cm^−1^ and new peaks appeared at 1461, 1384, and 842 cm^−1^, which were attributed to the -CH_2_CH_2_NH- moiety, indicating that chitosan/PEI cationic copolymers were successfully grafted on the surface of magnetic gelatin^15^.

Thermogravimetric analysis (TGA) was employed to study the thermal stability of magnetic composite (Fig. 2b). As shown in the TG plot, the magnetic composite supported a 3.8% weight loss at approximately 35–100 °C, which was ascribed to the removal of physically adsorbed water. During the heating procedure from 110 to 550 °C, the decrease in the weight of the magnetic gelatin was approximately 15.7%, which demonstrated the content of the gelatin coating. For the magnetic composite, there were two different types of TG curves in the 110–550 °C regions, implying the degradation of chitosan/PEI copolymer and gelatin, respectively. In the DTA plot of the magnetic composites, exothermic peaks (286 and 353 °C) were shifted to 325 and 382 °C, respectively, and a new peak was observed at 510 °C, implying the formation of chitosan/PEI-grafted magnetic gelatin.

X-ray diffraction (XRD) pattern in the 2θ range of 20–80° were used to study the effect of modification procedure on the magnetism (Fig. 2c). Six characteristic peaks were showed for Fe_3_O_4_ (2θ = 30.2^°^, 35.6^°^, 43.1^°^, 53.5^°^, 57.2^°^, and 62.8^°^), and the peak positions could be indexed to (220), (311), (400), (422), (511) and (440) (JCPDS Card: 19–629). The results demonstrated that the proposed modification did not adversely change the XRD phase of magnetic materials. Magnetization curves of the resulting polymers were showed in Fig. 2d at the room temperature. Due to the coating of chitosan/PEI cationic copolymers, the saturation magnetization of chitosan/PEI-grafted magnetic gelatin (41 emu g^−1^) was lower than that of magnetic gelatin (60 emu g^−1^). This feature provided an easy and efficient avenue for recovering the magnetic composites from aqueous solution under an external magnetic field. Furthermore, further studies are being undertaken by employing electromagnetic induction system to control the recovering of the magnetic composite and its application in the water purifiers. The above results indicated that chitosan/PEI-grafted magnetic gelatin with a high adsorption capacity and saturation magnetization has been formed in this study.

### Binding studies of the chitosan/PEI-grafted magnetic gelatin

**Figure 3:**
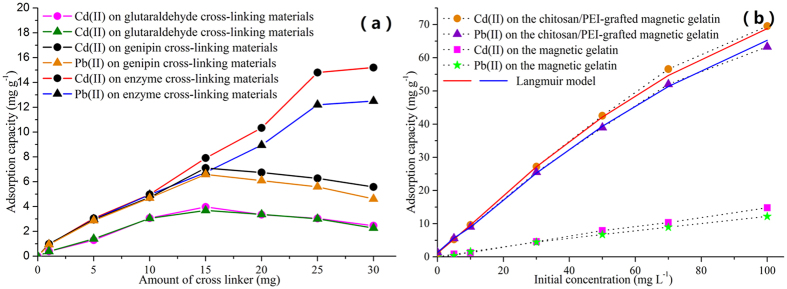
(**a**) Effect of different cross linkers on the adsorption capacity. (**b**) Adsorption isotherms of Pb(II) and Cd(II) on the magnetic gelatin and chitosan/PEI-grafted magnetic gelatin using TGase as the cross-linker, respectively.

The adsorption capacity is the essential property of magnetic composite as sorbents in the removal of heavy metals. To verify the novelty of the magnetic sorbents, magnetic gelatin was prepared using glutaraldehyde, genipin and TGase as the cross linkers, respectively. It is shown in Fig. 3a that the adsorption capacity of magnetic gelatin using genipin as the cross-linker was the same as that using TGase as the cross linker at low concentrations. However, the adsorption capacity of magnetic gelatin using genipin as the cross-linker showed a significant decrease at high concentration. The reason is that the intramolecular or intermolecular covalent bonds were formed and the polymer network density was increased with the increase of cross-linker. Thus, many active sites were easily embedded in the polymer matrix, which can decrease the adsorption capacity of sorbents. Due to the substrate specificity, cross-linking by enzymes could remain many of the amino groups and spatial structure of gelatin molecule, which was beneficial for the exposure of active sites and the further modification. After grafting with chitosan/PEI cationic copolymers, the adsorption capacity of the magnetic composite was greatly enhanced (66.1 mg g^−1^ and 65.2 mg g^−1^) (Fig. 3b). Furthermore, microbial TGase is environmentally friendly, available in large quantities and easier to handle, which has stimulated research on it for a variety of applications, especially in the food industry^20^. The 2,4,6-trinitrobenzenesulfonic acid reaction was also used to study the change of amino groups. It was shown that the amino groups of magnetic gelatin using transglutaminase as a cross-linker was 0.031 mmol g^−1^, which was more than that of magnetic gelatin using genipin as a cross-linker (0.014 mmol g^−1^). After grafting of chitosan-PEI cationic copolymers, the amino groups were increased to 0.117 mmol g^−1^. It was shown transglutaminase as a cross-linker and the modification of cationic copolymers were proposed to increase the number of amino groups and then improve the removal efficiency of sorbents.

**Figure 4:**
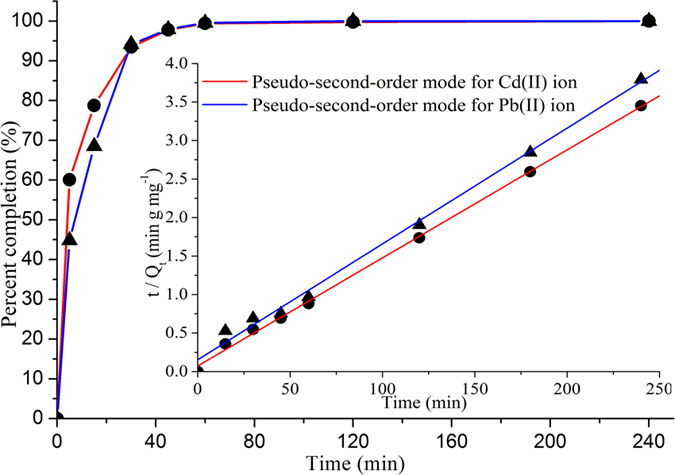
Treatment capacity of chitosan/PEI-grafted magnetic gelatin for Pb(II) and Cd(II) with initial concentrations at 200 ng mL^−1^.

The removal efficiency at a low concentration is the essential for the purification of drinking water using magnetic composites. In general, the concentration of heavy metals is no more than 200 ng mL^−1^ in real drinking water. Therefore, 10 mg of magnetic composite was mixed with a heavy metal solution and the initial concentration was fixed at 200 ng mL^−1^, while varying the volume from 0.1 to 1 L. The limits set by the WHO were 10 ng mL^−1^ for Pb(II) and 5 ng mL^−1^ for Cd(II) for drinking water^22^. The results indicated that 10 mg of chitosan/PEI-grafted magnetic gelatin could purify approximately 0.8 L of drinking water spiked with 200 ng mL^−1^ of Pb(II) or 0.4 L of drinking water spiked with 200 ng mL^−1^ of Cd(II) (Fig. 4). It was shown that chitosan/PEI-grafted magnetic gelatin could be used to remove heavy metals in different drinking water by adjusting the amount of magnetic composite.

Furthermore, the adsorption isotherms can be used to study how heavy metals interact with the chitosan/PEI-grafted magnetic gelatin. The values of these parameters are summarized in Table 1. The experimental data were good fit with the Langmuir model, which showed that the binding of heavy metals on the magnetic composites was controlled monolayer adsorption. The Q_max_ values of the PEI-grafted gelatin sponge were 341 mg g^−1^ for Pb(II) and 321 mg g^−1^ for Cd(II), respectively. Furthermore, the essential characteristics of the Langmuir model, named as equilibrium parameter (R_L_), was further studied. The equilibrium parameters for both of the test ions on the magnetic composite were in the range of 0.42–0.52, which showed that the experimental conditions were favorable for the adsorption of heavy metals.

**Figure 5:**
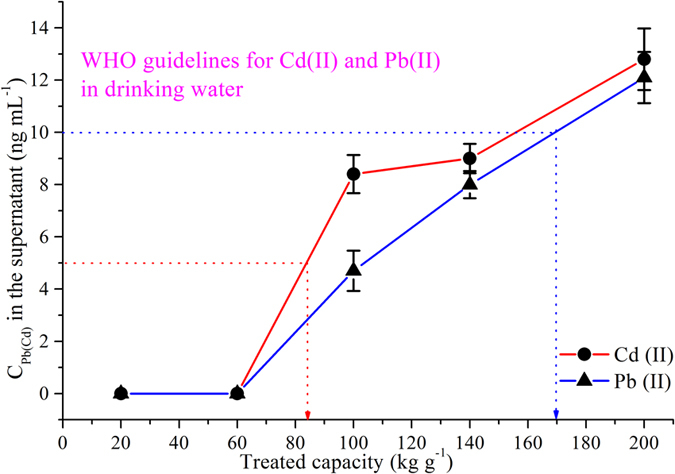
Rebinding kinetic behavior of Pb(II) and Cd(II) on the chitosan/PEI-grafted magnetic gelatin. Inset: the pseudo-second-order mode for the adsorption of Pb(II) and Cd(II).

The adsorption kinetics is very important characterization for the application of chitosan/PEI-grafted magnetic gelatin. It can be seen from Fig. 5 that the magnetic composites provided the rapid adsorption of heavy metals (45 min), because of the large surface area of the nanomaterials with the monolayer structure. The mechanism of the adsorption kinetics was further investigated by different models and the results are shown in Table 2. The calculated values of the adsorption capacity (Q_e,calc_) were the same as the experimental values (Q_e,exp_) and the correlation coefficients (R^2^) of the pseudo-second-order kinetic equation were all above 0.995. Thus, the adsorption of heavy metals on the magnetic composite met the pseudo-second-order kinetic model, which was the rate-limiting step of the adsorption process^10^. As seen in Table S1 (Supporting Information), the adsorption capacities of the sorbents using chitosan or gelatin as the monomer were low, and the commonly used cross-linker was glutaraldehyde. Thus, these sorbents cannot be used to remove heavy metals from drinking water because of the low adsorption capacities and the toxicity of glutaraldehyde. The above results indicated that the high adsorption capacity, modification of the cationic copolymer and rapid magnetic separation of the magnetic composite were in favor of a high adsorption capacity and mass transfer.

### Effects of the experimental conditions on the removal of heavy metals

Due to the complexity and variability of natural drinking water, it is highly important to investigate the adsorption capabilities of chitosan/PEI-grafted magnetic gelatin under various experimental conditions. First, the pH value affected not only the forms of heavy metals in aqueous solution, but also the charge characteristics of the functional groups. Thus, the pH value of heavy metal solution was the key factor for the removal efficiency of sorbents. As shown in Fig. S4 (Supporting Information), the adsorption capacities showed maximums when the pH value was changed from 6.0 to 7.0, with a decrease at lower and higher pH values. Large amounts of H^+^ in acid solution could compete with the target ions to decrease the adsorption capacity of magnetic composites, while hydroxide in basic solution can precipitate metal ions^23^. Because the pH values of practical drinking water were changed to be in the pH range of 6.5 to 7.5, real water samples could be directly mixed with the magnetic composites to remove heavy metals.

The effect of temperature on the removal efficiency of chitosan/PEI-grafted magnetic gelatin was studied in this study. We tried to investigate the temperature of water purification in spring/autumn (277 K), winter (293 K) and summer (303 K) and thermodynamic parameters were calculated, such as the enthalpy (ΔH°), entropy (ΔS°) and Gibbs energy (ΔG°, Table 3)^24^. The negative values of ΔG° suggested that the adsorption of heavy metals was a thermodynamically favorable binding procedure in the studied range of 277–303 K. The positive values of ΔS° indicated that the increase of the randomness at the solid-liquid interface. The positive values of ΔH° demonstrated the heavy metals adsorption onto magnetic composites is endothermic in nature. These parameters could be used to provide practical guidance for the application of magnetic composites.

**Figure 6:**
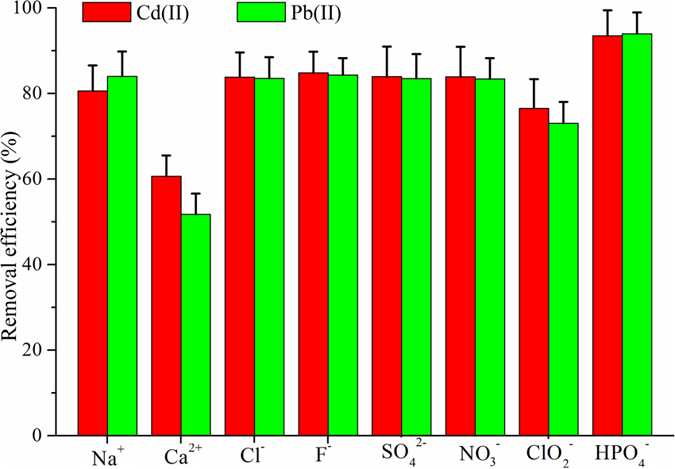
Effects of coexisting ions on the adsorption capacity of Pb(II) and Cd(II) on the chitosan/PEI-grafted magnetic gelatin. The concentrations of Pb(II) and Cd(II) were 200 ng mL^−1^. The concentration of cations was 450 μg mL^−1^. The concentration of ClO_2_^−^ was 0.7 μg mL^−1^ and another anions were 250 μg mL^−1^ (Based on the regulatory limits of Drinking Water Standards of China, 2006).

The active sites of magnetic composites can also be used to adsorb the common ions in drinking water, which would decrease the practical value of sorbents (Fig. 6). Therefore, we further investigated the effect of interference ions on the removal efficiency, such as monovalent cations (Na^+^), divalent cations (Ca^2+^ as a water hardness index) and anions (F^−^, Cl^−^, NO_3_^−^, SO_4_^2−^, ClO_2_^−^ and HPO_4_^−^). The influence of ClO_2_^−^ was especially important, because sodium hypochlorite often consumed in the drinking water treatment, and the chlorine demand was found to be approximately 0.5 mg L^−1 ^25. The results indicated that Ca^2+^ led to decreased trend of the removal efficiency of heavy metals, due to different electric charges and hydration energies^10^. For the coexisting anions, ClO_2_^−^ also exhibited a prominent effect on the removal performance of magnetic composite, which might compete with the cationic charges of PEI/chitosan cationic copolymers and then decrease the active sites of the magnetic composite. It is interesting to note that chitosan and PEI has been known for their antibacterial activity against a wide range of microorganisms^26^,^27^. Thus, the amount of sodium hypochlorite could be decreased due to the antibacterial effectiveness of magnetic composites. This property was beneficial for the practical application of sorbents while decreasing the highly toxic chlorination disinfection byproducts in drinking water.

### Stability and regeneration

In the practical application of magnetic composites, the stability and reuse are economic necessity. The chitosan/PEI-grafted magnetic gelatin was stored in aqueous solution at room temperature when not in use. The adsorption capacity was not changed under the same operational condition after 30 d of storage, demonstrating the excellent stability over time. Since the adsorption of heavy metals on the sorbent was a reversible process, the magnetic composite could be introduced into the heavy metals solutions and then taken out for regeneration using an acetic acid solution (0.1 mmol L^−1^). Although the efficiency decreased with an increasing number of cycles, over 89% of efficiency was obtained in the fifth adsorption-desorption cycles, demonstrating that the magnetic composites was cost-effective (Fig. S5, Supporting Information). After five cycles, the magnetic intensity did not decrease and the magnetic composites could be rapidly separated from solution. In addition, there was no leaching of iron ions from the composite into the solution phase, implying that the dissolution of the magnetic core under the stated experimental conditions was negligible.

## Conclusion

By using TGase as a cross linker, chitosan/PEI-grafted magnetic gelatin was prepared to remove heavy metals from drinking water. Due to the substrate specificity, cross-linking by enzymes could remain many of the amino groups needed for removal of the heavy metals in water. After further modification with chitosan-PEI cationic copolymers, the number of amino groups was greatly increased and the adsorption capacity was enhanced. Meanwhile, this material was an environmental friendly sorbent with magnetic susceptibility and excellent water compatibility. It is expected that chitosan/PEI-grafted magnetic gelatin has broad applications for the removal of heavy metals.

## Methods

### Synthesis of magnetic gelatin

Gelatin solution (60 mg mL^−1^) was prepared by swelling gelatin powder in an aqueous solution and dissolving at 55 °C. Then, 1.3 g of anhydrous ferric chloride and 0.75 g of ferrous sulfate were added. Five milliliters of ammonia solution was added dropwise into the solution. After 6 h, the resulting particles were magnetically collected and then washed with distilled water and ethanol, respectively. After dispersion in an aqueous solution, 25 mg of TGase was introduced to the mixture and the reaction was performed at room temperature for 8 h. Finally, the magnetic gelatin was magnetically collected, washed with distilled water, and then dried in a vacuum oven.

### Synthesis of chitosan/PEI-grafted magnetic gelatin

Preparation of chitosan/PEI-grafted magnetic gelatin is shown in Fig. 1. Chitosan was firstly oxidized by potassium periodate to obtain the dialdehyde chitosan, which was prepared based on the literature with a slight modification^28^. Briefly, chitosan was dissolved in acetic acid (0.2 mol L^−1^) to a final concentration of 60 mg mL^−1^. Then, 0.49 mg of potassium periodate was added into the mixture with continuous stirring in the dark for 24 h. After adjusting the pH value to 7.5, the resulting product was purified by extensive dialysis (molecular weight cut-off, MWCO 10,000) against deionized water for 4 days.

Subsequently, a cationic copolymer was synthesized based on a reductive amination reaction between PEI and chitosan. Fifty-six milligrams of dialdehyde chitosan was dissolved in 5 mL of water and then slowly added into 0.1 mol L^−1^ of borate buffer (pH = 11) spiked with 450 mg of PEI. After incubation for 24 h, 30 mg of NaBH_4_ was added and the resulting solution was further incubated for 48 h. The reduction could be repeated several times until the resulting solution became yellow, followed by extensive dialysis (MWCO 5,000) against deionized water for 2 days. After filtering, the product was obtained by using freeze-drying technology.

Finally, chitosan/PEI cationic copolymers were grafted onto the surface of magnetic gelatin^29^. 3,3′-dithiodipropionic acid solution (0.2 mol L^−1^) was prepared by using aqueous dimethylformamide and then mixed with a 0.1 mol L^−1^ of EDC solution. The mixture was stirred at 40 °C for 1 h and then slowly added dropwise into the aqueous solution (pH = 5.0) containing the magnetic gelatin. After incubation at 40 °C for 24 h, the resulting product was magnetically collected and redispersed in an aqueous solution containing 0.5 mmol of chitosan/PEI cationic copolymers, 0.5 mmol EDC and 0.5 mmol NHS. The resulting solution was incubated at 40 °C for 24 h with continuous shaking. The chitosan/PEI-grafted magnetic gelatin was obtained by washing with water and magnetic separation.

### Adsorption Studies

Batch experiments were performed to investigate the binding behavior and the kinetics procedure of sorbents. Briefly, chitosan/PEI-grafted magnetic gelatin was added into the aqueous solutions spiked with different concentrations of heavy metal (1 to 100 mg L^−1^). After shaking at 400 rpm for 2 h, the composites were recovered by magnetic separation, and the target ion in supernatant was determined using an inductively coupled plasma-mass spectrometry (ICP-MS, Agilent 7500, Agilent, USA). Kinetic studies were the same as the adsorption experiments, except for the different time intervals.

Furthermore, the effects of the temperature, pH value and coexisting anions/cations of drinking water on the removal efficiency were studied. The removal efficiency was calculated based on the different amounts of target ions with and without the treatment of magnetic composites. The pH values were changed from 4.0 to 10.0 and the temperature were set at 277 K, 293 K and 303 K, which were the same as the environmental temperatures in spring/autumn, winter and summer, respectively. Moreover, based on the regulatory limits of drinking water standards of China (2006), the concentration of cations (Na^+^ and Ca^+^) was 450 μg mL^−1^, ClO_2_^−^ was 0.7 μg mL^−1^ and another anions (Cl^−^, F^−^, SO_4_^2−^, NO_3_^−^ and HPO_4_^−^) were 250 μg mL^−1^. For regeneration, the particles were eluted by 10 mL of HCl solution (10 mmol L^−1^) and then washed with water. Regenerated magnetic composites were used for adsorption in successive cycles.

## Additional Information

**How to cite this article:** Li, B. *et al*. Environmentally friendly chitosan/PEI-grafted magnetic gelatin for the highly effective removal of heavy metals from drinking water. *Sci. Rep.* **7**, 43082; doi: 10.1038/srep43082 (2017).

**Publisher's note:** Springer Nature remains neutral with regard to jurisdictional claims in published maps and institutional affiliations.

## Supplementary Material

Supplementary Information

## Figures and Tables

**Table 1:** Isotherm constants for adsorption of Pb(II) and Cd(II) onto the chitosan/PEI-grafted magnetic gelatin.

Target ions	Langmuir model				Freundlich model		
	Q_max_ (mg g^−1^)	K_L_ (L mg^−1^)	R_L_	R^2^	K_F_ (mg g^−1^)	n	R^2^
Cd(II)	53.94	0.0218	0.46	0.9923	1.35	1.19	0.9831
	321.9	0.0031	0.50	0.9948			
Pb(II)	28.25	0.049	0.42	0.9925	1.49	1.25	0.9890
	341.7	0.0027	0.52	0.9924			

**Table 2:** Kinetic constants for the adsorption of Pb(II) and Cd(II) onto the chitosan/PEI-grafted magnetic gelatin.

Test metal ions	Pseudo-First order			Pseudo-Second order			Intra-particle diffusion model		
	k_1_ (min^−1^)	Q_e,calc*_ (mg g^−1^)	R^2^	k_2_ (g mg^−1 ^min^−1^)	Q_e,calc*_ (mg g^−1^)	R^2^	k_i_ (mg g^−1 ^min^−1/2^)	C	R^2^
Pb(II)	0.023	57.90	0.9695	0.00082	64.52	0.9968	2.0578	25.739	0.7674
Cd(II)	0.018	50.32	0.9854	0.00073	63.70	0.9964	2.0848	24.059	0.7674

^*^Q_e_ denotes the adsorbed amount of Pb(II) and Cd(II) at the equilibrium concentration and Q_e,calc_ is the calculated value of Q_e_ based on the kinetic model. The experimental values of Q_e_ of Pb(II) and Cd(II) are 61.31 mg g^−1^ and 60.46 mg g^−1^, respectively.

**Table 3:** Thermodynamic parameters for the adsorption of Pb(II) and Cd(II) onto the chitosan/PEI-grafted magnetic gelatin.

Target ions	Temperature (K)	Thermodynamic parameters		
		△G^0^ (kJ mol^−1^)	△H^0^ (kJ mol^−1^)	△S^0^ (J mol^−1 ^k^−1^)
Cd(II)	277	−3.61	3.32	13.03
	293	−3.81		
	303	−3.94		
Pb(II)	277	−1.07	2.32	3.88
	293	−1.13		
	303	−1.17		

## References

[b1] Li Y. H. . Adsorption thermodynamic, kinetic and desorption studies of Pb^2+^ on carbon nanotubes. Water Res 39, 605–609 (2005). 15707633 10.1016/j.watres.2004.11.004

[b2] Levallois P. . The impact of drinking water, indoor dust and paint on blood lead levels of children aged 1–5 years in Montréal (Québec, Canada). J Expo Sci Env Epid 24, 185–191 (2014). 10.1038/jes.2012.129 PMC3929778 23361441

[b3] Flora G., Gupta D. & Tiwari A. Toxicity of lead: a review with recent updates. Interdiscipl Toxicol 5, 47–58 (2012). 10.2478/v10102-012-0009-2 PMC3485653 23118587

[b4] Lanphear B. P. . Low-level environmental lead exposure and children’s intellectual function: an international pooled analysis. Environ Health Persp 113, 894–899 (2005). 10.1289/ehp.7688 PMC1257652 16002379

[b5] Deliyanni E. A., Kyzas G. Z., Triantafyllidis K. S. & Matis K. A. Activated carbons for the removal of heavy metal ions: A systematic review of recent literature focused on lead and arsenic ions. Open Chem 13, 699–708 (2015).

[b6] Saif M. J., Zia K. M., Usman M., Hussain A. I. & Chatha S. A. S. Removal of Heavy Metals by Adsorption onto Activated Carbon Derived from Pine Cones of Pinus roxburghii. Water Environ Res 87, 291–297 (2015). 26462072 10.2175/106143015X14212658613433

[b7] Azamat J., Khataee A. & Joo S. W. Removal of heavy metals from water through armchair carbon and boron nitride nanotubes: a computer simulation study. RSC Adv 5, 25097–25104 (2015).

[b8] Xie Y. . Mussel inspired functionalization of carbon nanotubes for heavy metal ion removal. RSC Adv 5, 68430–68438 (2015).

[b9] Gu X., Yang Y., Hu Y., Hu M. & Wang C. Fabrication of graphene-based xerogels for removal of heavy metal ions and capacitive deionization. ACS Sustain Chem Eng 3, 1056–1065 (2015).

[b10] Kim E. J., Lee C. S., Chang Y. Y. & Chang Y. S. Hierarchically structured manganese oxide-coated magnetic nanocomposites for the efficient removal of heavy metal ions from aqueous systems. Acs Appl Mater Inter 5, 9628–9634 (2013). 10.1021/am402615m 24028422

[b11] Wang W. . Hydrothermal synthesis of hierarchical core–shell manganese oxide nanocomposites as efficient dye adsorbents for wastewater treatment. RSC Adv 5, 56279–56285 (2015).

[b12] Godwin H. . Nanomaterial categorization for assessing risk potential to facilitate regulatory decision-making. ACS nano 9, 3409–3417 (2015). 25791861 10.1021/acsnano.5b00941

[b13] Simate G. S., Iyuke S. E., Ndlovu S., Heydenrych M. & Walubita L. F. Human health effects of residual carbon nanotubes and traditional water treatment chemicals in drinking water. Environ Int 39, 38–49 (2012). 22208741 10.1016/j.envint.2011.09.006

[b14] Khan K. . Manganese exposure from drinking water and children’s academic achievement. Neurotoxicology 33, 91–97 (2012). 22182530 10.1016/j.neuro.2011.12.002 PMC3282923

[b15] Jiang W. . Facile fabrication of magnetic chitosan beads of fast kinetics and high capacity for copper removal. Acs Appl Mater Inter 6, 3421–3426 (2014). 10.1021/am405562c 24524391

[b16] Bi X., Lau R. J. & Yang K.-L. Preparation of ion-imprinted silica gels functionalized with glycine, diglycine, and triglycine and their adsorption properties for copper ions. Langmuir 23, 8079–8086 (2007). 17567056 10.1021/la7008072

[b17] Chen G., Qiao C., Wang Y. & Yao J. Synthesis of magnetic gelatin and its adsorption property for Cr (VI). Ind Eng Chem Res 53, 15576–15581 (2014).

[b18] Gyananath G. & Balhal D. Removal of lead (II) from aqueous solutions by adsorption onto chitosan beads. Cell Chem Technol 46, 121–124 (2012).

[b19] Cui L., Jia J., Guo Y., Liu Y. & Zhu P. Preparation and characterization of IPN hydrogels composed of chitosan and gelatin cross-linked by genipin. Carbohydr Polym 99, 31–38 (2014). 24274476 10.1016/j.carbpol.2013.08.048

[b20] Spolaore B. . Local unfolding is required for the site-specific protein modification by transglutaminase. Biochemistry-US 51, 8679–8689 (2012). 10.1021/bi301005z 23083324

[b21] Li W.-M., Liu D.-M. & Chen S.-Y. Amphiphilically-modified gelatin nanoparticles: Self-assembly behavior, controlled biodegradability, and rapid cellular uptake for intracellular drug delivery. J Mater Chem. 21, 12381–12388 (2011).

[b22] Cao C.-Y., Qu J., Wei F., Liu H. & Song W.-G. Superb adsorption capacity and mechanism of flowerlike magnesium oxide nanostructures for lead and cadmium ions. Acs Appl Mater Inter 4, 4283–4287 (2012). 10.1021/am300972z 22812446

[b23] Kosa S. A., Al-Zhrani G. & Salam M. A. Removal of heavy metals from aqueous solutions by multi-walled carbon nanotubes modified with 8-hydroxyquinoline. Chem Eng J 181, 159–168 (2012).

[b24] Li X., Li Y., Zhang S. & Ye Z. Preparation and characterization of new foam adsorbents of poly (vinyl alcohol)/chitosan composites and their removal for dye and heavy metal from aqueous solution. Chem Eng J 183, 88–97 (2012).

[b25] Regli S. . Estimating Potential Increased Bladder Cancer Risk Due to Increased Bromide Concentrations in Sources of Disinfected Drinking Waters. Environ Sci Tech 49, 13094–13102 (2015). 10.1021/acs.est.5b03547 26489011

[b26] Barros J. . Antibiofilm and Antimicrobial Activity of Polyethylenimine: An Interesting Compound for Endodontic Treatment. J Contemp Dent Pract 16, 427–432 (2014). 10.5005/jp-journals-10024-1701 26323443

[b27] Gylienė O. . Correlation between the sorption of dissolved oxygen onto chitosan and its antimicrobial activity against Esherichia coli. Carbohydr Polym 131, 218–223 (2015). 26256178 10.1016/j.carbpol.2015.05.068

[b28] Ping Y. . Chitosan-graft-(PEI-β-cyclodextrin) copolymers and their supramolecular PEGylation for DNA and siRNA delivery. Biomaterials 32, 8328–8341 (2011). 21840593 10.1016/j.biomaterials.2011.07.038

[b29] Li Z. T. . Chitosan-graft-polyethylenimine with improved properties as a potential gene vector. Carbohydr Polym 80, 254–259 (2010).

