# Supplementary material for: Environmentally friendly chitosan/PEI-grafted magnetic gelatin for the highly effective removal of heavy metals from drinking water
Source: Sci Rep. 2017 Feb 22;7:43082. doi: 10.1038/srep43082 (PMC5320531; doi:10.1038/srep43082)

# Environmentally friendly chitosan/PEI-grafted magnetic gelatin for the highly effective removal of heavy metals from drinking water

Bingbing Li^1^; Feng Zhou^1^; Kai Huang^1^; Yipei Wang^2^; Surong Mei^1^; Yikai Zhou^1^;Tao Jing^1*^

^1^ State Key Laboratory of Environment Health (Incubation), Key Laboratory of Environment and Health, Ministry of Education, Key Laboratory of Environment and Health (Wuhan), Ministry of Environmental Protection, School of Public Health, Tongji Medical College, Huazhong University of Science and Technology, #13 Hangkong Road, Wuhan, Hubei, 430030, China

^2^ Institute of Environmental Pollution and Health, School of Environmental and Chemical Engineering, Shanghai University, Shanghai 200444, China.

* **Corresponding Author:**

**Tao Jing**, E-mail: jingtao@hust.edu.cn

Address: School of Public Health, Tongji Medical College, Huazhong University of Science and Technology, #13 Hangkong Road, Wuhan, Hubei, 430030, China

Tel: +86(27)-83552611

Fax: +86(27)-83657765

**Table S1** Comparison of the proposed sorbent with other sorbents reported in the literatures using chitosan or gelatin as the monomer

| Sorbents | Cross-linker | Adsorption capacities  (mg g^-1^) | Equilibrium time  (min) | Ref. |
| --- | --- | --- | --- | --- |
| Plasma electrolytic oxidation/Chitosan nanofiber membrane | Glutaraldehyde | 232 for Cd(II)  214 for Pb(II) | 120 | 1 |
| Magnetic chitosan/graphene oxide composites | Glutaraldehyde | 76.94 for Pb(II) | 40 | 2 |
| Polyaniline grafted chitosan | Glutaraldehyde | 14.33 for Cd(II)  16.07 for Pb(II) | 45 | 3 |
| Chitosan/Sulfydryl functionalized  graphene oxide composites | Glutaraldehyde | 177 for Cd(II) | 10 | 4 |
| Chitosan-stabilized nano zero-valent iron | Without cross-linker | 124.74 for Cd(II) | 180 | 5 |
| Pollen-chitosan microcapsules | Glutaraldehyde | 65.98 for Cd(II) | 240 | 6 |
| Chitosan/biomass/gelatin bead | Glutaraldehyde | 35 for Pb(II) | 120 | 7 |
| Chitosan-clay composite beads | Epichlorohydrin | 72.31 for Cd(II) | 120 | 8 |
| Magnetic hydroxypropyl chitosan/oxidized multiwalled carbon nanotubes | Glutaraldehyde | 116.3 for Pb(II) | 60 | 9 |
| Porous chitosan/gelatin foams | Glutaraldehyde | 70 for Pb(II) | 240 | 10 |
| Chitosan/PEI-grafted magnetic gelatin | Transglutaminase | 342 for Cd(II)  341 for Pb(II) | 45 | This work |

**References:**

1 . Aliabadi, M., Irani, M., Ismaeili, J., Piri, H. & Parnian, M. J. Electrospun nanofiber membrane of PEO/Chitosan for the adsorption of nickel, cadmium, lead and copper ions from aqueous solution. *Chem Eng J* **220**, 237-243 (2013).

2. Fan, L., Luo, C., Sun, M., Li, X. & Qiu, H. Highly selective adsorption of lead ions by water-dispersible magnetic chitosan/graphene oxide composites. *Colloid Surface B* **103**, 523-529 (2013).

3 . Karthik, R. & Meenakshi, S. Removal of Pb (II) and Cd (II) ions from aqueous solution using polyaniline grafted chitosan. *Chem Eng J* **263**, 168-177 (2015).

4. Li, X. *et al.* Studies of heavy metal ion adsorption on Chitosan/Sulfydryl-functionalized graphene oxide composites. *J Colloid Inter Sci* **448**, 389-397 (2015).

5 . Lu, H. *et al.* Effective removal of cadmium ions from aqueous solution using chitosan-stabilized nano zero-valent iron. *Desalin Water Trea* **56**, 256-265 (2015).

6 . Sargın, İ., Kaya, M., Arslan, G., Baran, T. & Ceter, T. Preparation and characterisation of biodegradable pollen–chitosan microcapsules and its application in heavy metal removal. *Bioresource Technol* **177**, 1-7 (2015).

7 . Shaker, M. A. Equilibrium, kinetics and thermodynamics studies of chitosan-based solid phase nanoparticles as sorbent for lead (II) cations from aqueous solution. *Mater Chem Phys* **162**, 580-591 (2015).

8. Tirtom, V. N., Dinçer, A., Becerik, S., Aydemir, T. & Çelik, A. Comparative adsorption of Ni (II) and Cd (II) ions on epichlorohydrin crosslinked chitosan–clay composite beads in aqueous solution. *Chem Eng J* **197**, 379-386 (2012).

9. Wang, Y. *et al.* The removal of lead ions from aqueous solution by using magnetic hydroxypropyl chitosan/oxidized multiwalled carbon nanotubes composites. *J Colloid Inter Sci* **451**, 7-14 (2015).

10. Zhang, Y. *et al.* Fabrication of chitosan/gelatin foams with ordered porous structures for use in drug release and metal ion adsorption. *RSC Adv* **4**, 33840-33847 (2014).

**Figure S1.** Transglutaminase (TGase)-mediated cross-linking of magnetic composites. Transamidation between protein-bound Gln and Lys residues leads to the formation of g-glutamyl-e-lysyl isopeptide bonds.


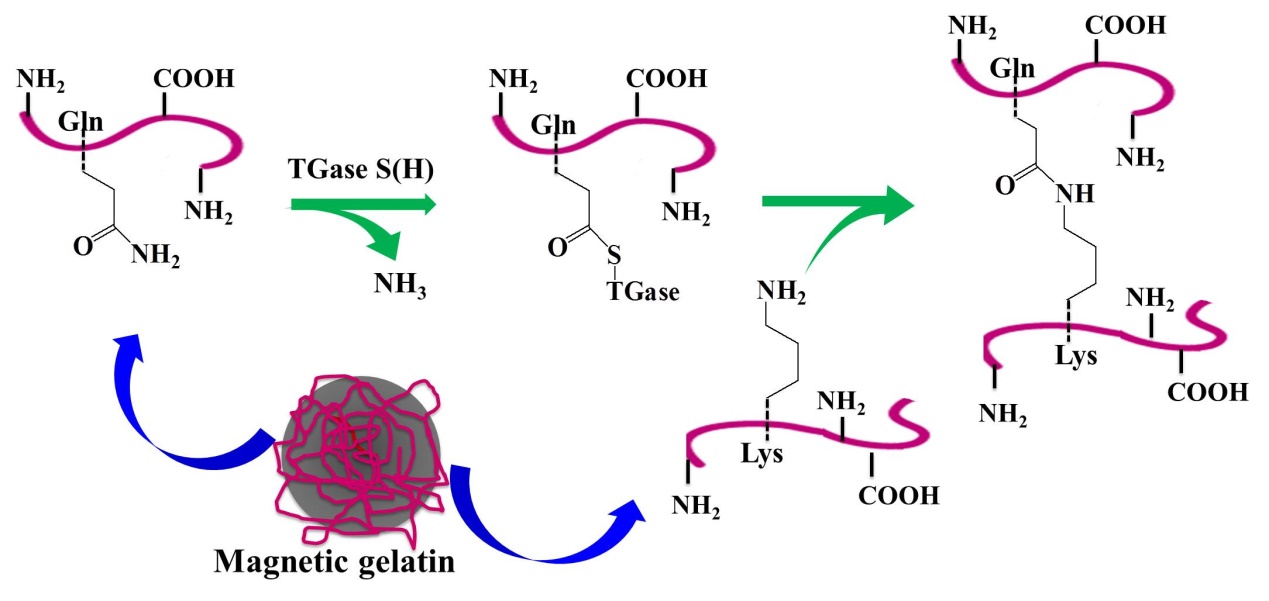


**Figure S2.** ^1^H NMR spectra of chitosan, dialdehyde chitosan and chitosan/PEI copolymer


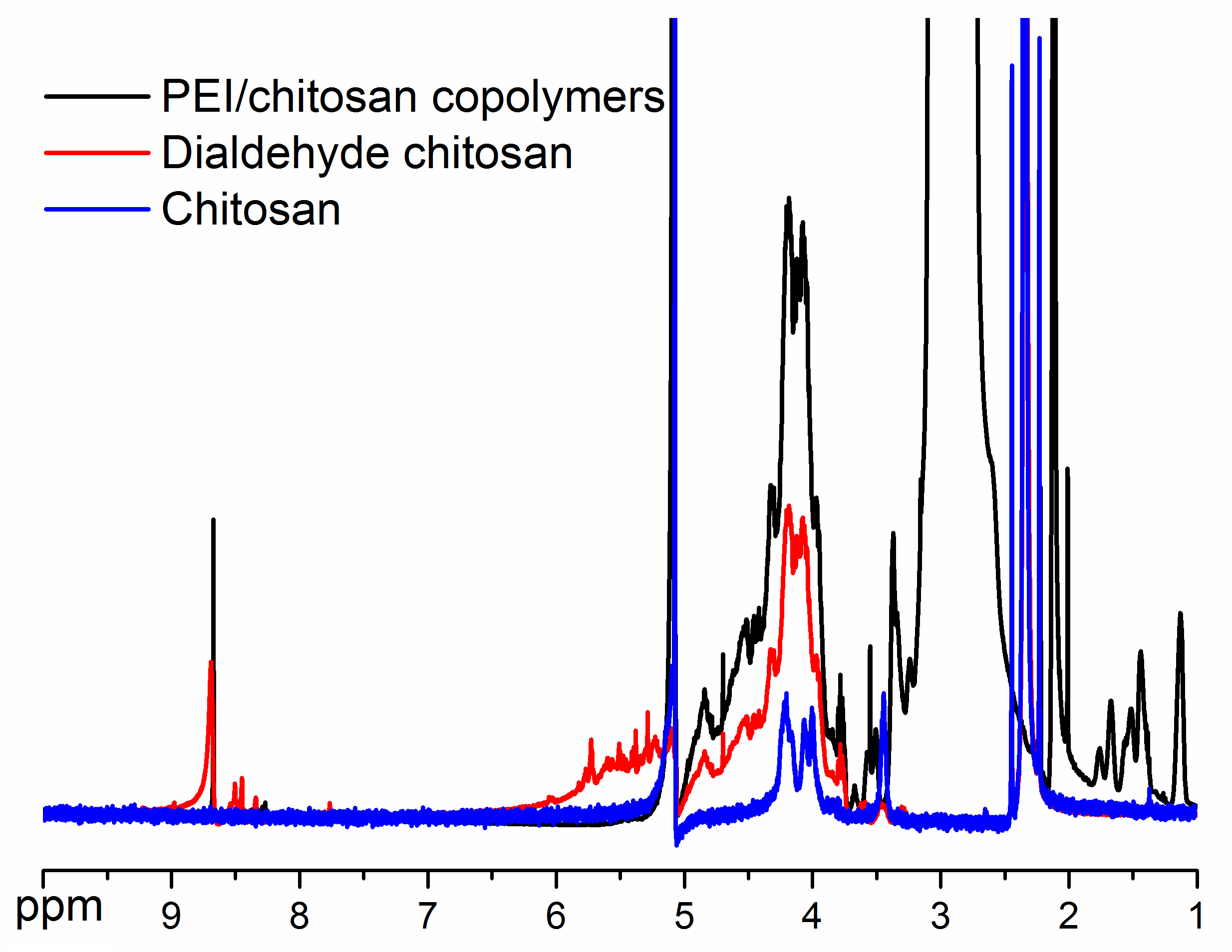


**Figure S3.** TEM image of magnetic gelatin (A) and chitosan/PEI-grafted magnetic gelatin (B)


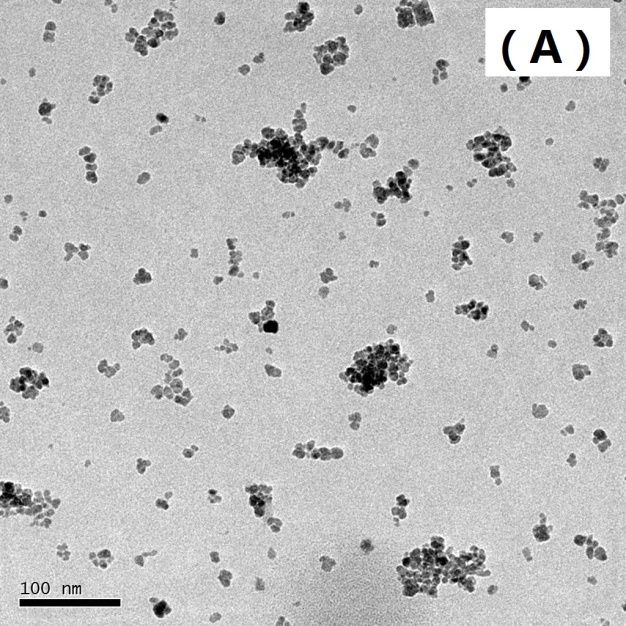

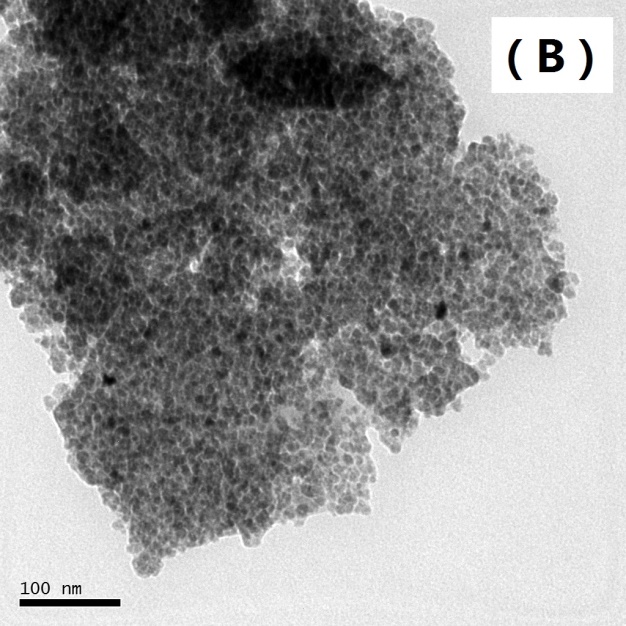


**Figure S4.** Effects of the pH on the adsorption capacities of Pb(II) and Cd(II) on the chitosan/PEI-grafted magnetic gelatin.


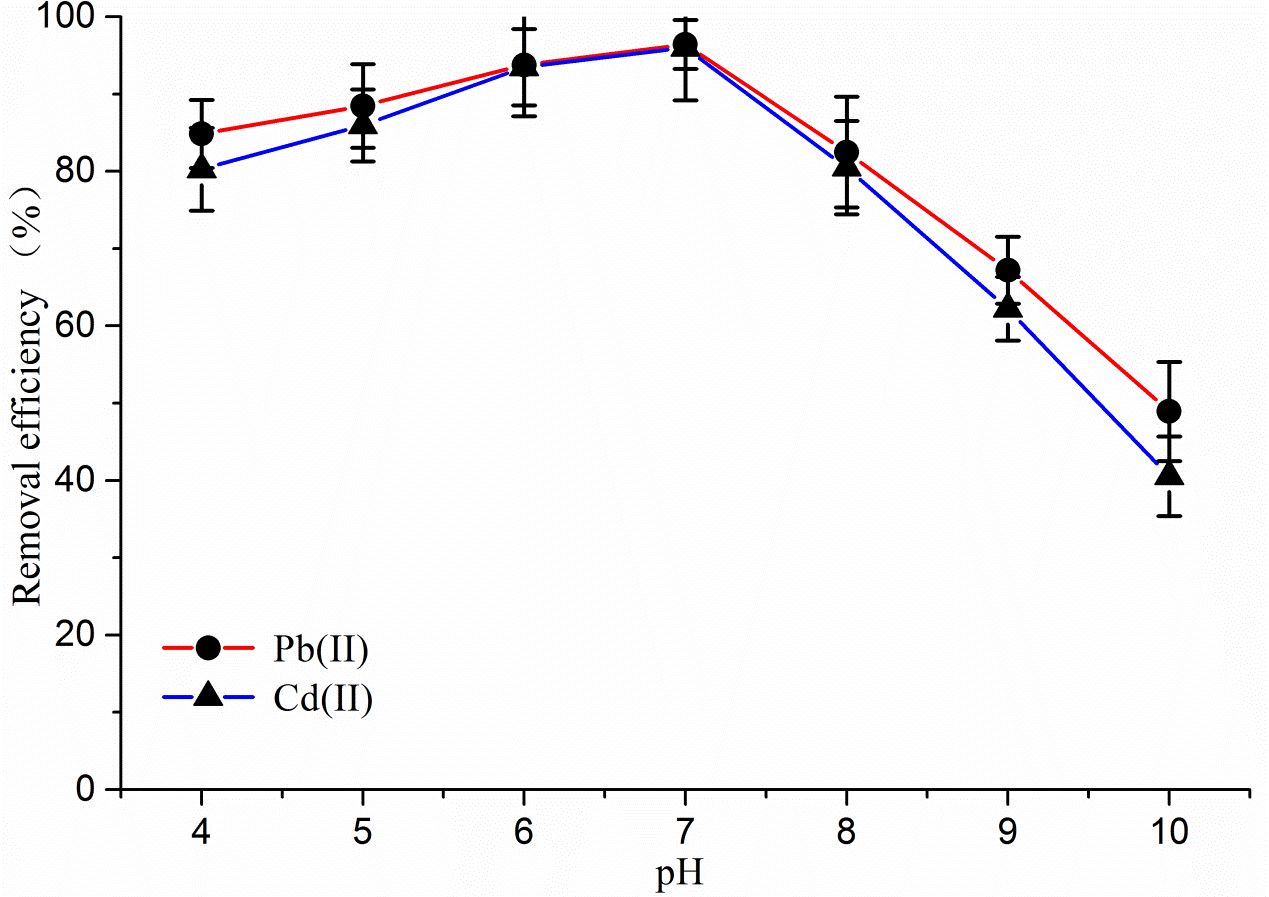


**Figure S5**. Regeneration studies of chitosan/PEI-grafted magnetic gelatin with five cycles. The initial concentrations of Pb(II) and Cd(II) were 200 ng mL^-1^. The contact time was 2 h for each cycle.


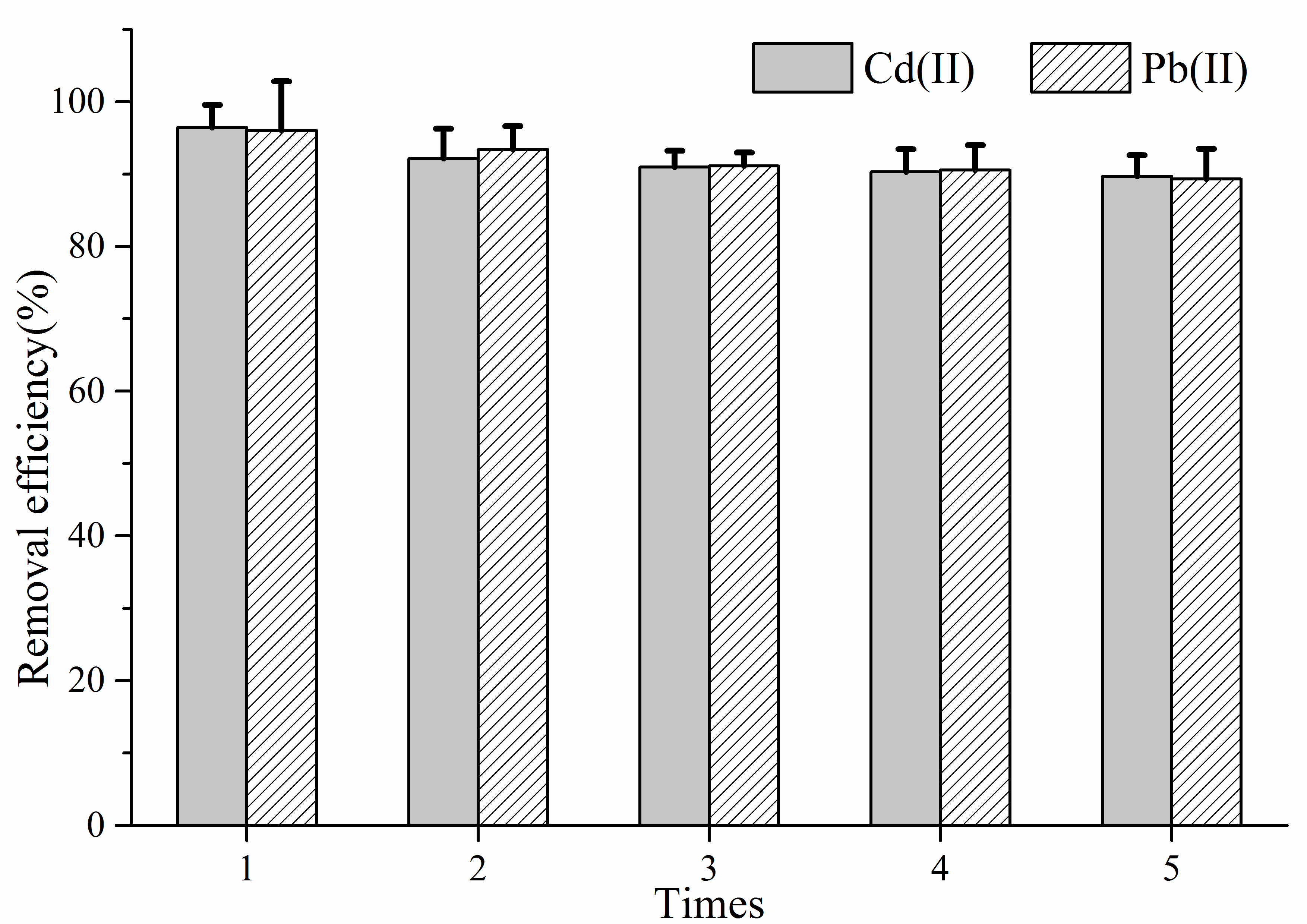

Supplement: Supplementary Information [file srep43082-s1.doc]
